# Institutional tuberculosis infection control in a rural sub-district in South Africa: A quality improvement study

**DOI:** 10.4102/phcfm.v11i1.1971

**Published:** 2019-06-26

**Authors:** Martha B. Mekebeb, Klaus von Pressentin, Louis S. Jenkins

**Affiliations:** 1 Department of Family and Emergency Medicine, Faculty of Health Sciences, Stellenbosch University, Cape Town, South Africa; 2 Mossel Bay Hospital, Garden Route District, South Africa; 3 George Hospital, Garden Route District, South Africa

**Keywords:** tuberculosis, infection prevention and control, quality improvement cycle, primary health care, district health services

## Abstract

**Background:**

Tuberculosis (TB) is a major global health challenge, and South Africa is one of the high-burden countries. A national TB infection control (TBIC) guideline has stipulated three areas of infection control at health facilities: work practice and administrative control, environmental control, and personal protection for health workers.

**Aim:**

The aim of this study was to identify the gaps and address the challenges in institutional TBIC.

**Setting:**

The district hospital and a primary health care clinic within the Mossel Bay sub-district in the Western Cape.

**Methods:**

According to the national TBIC draft guideline, a quality improvement cycle was used to evaluate and improve TBIC. Each facility had an existing infection and prevention control and occupational health and safety team, which were used as the audit teams.

**Results:**

A baseline assessment was followed by a set of interventions, which did not show a significant improvement in TBIC. The difference between the pre- and post-intervention TB screening rate was not statistically significant. An assessment of time interval between 101 patients presenting with TB symptoms and diagnosed with TB was 4 days at baseline and post-intervention. Most of the anticipated improvements were dependent on the health workers’ adherence to the local TBIC policies, which emerged as an unexpected finding.

**Conclusion:**

We found good managerial commitment reflected by the presence of various policies, guidelines, specific personnel and committees to deal with infection control in general. This study has created awareness about TBIC among staff and pointed out the complexity of health workers’ behaviour towards adhering to policies.

## Background

Tuberculosis (TB) is a major global health challenge. Globally, the incidence of new TB cases in 2015 was higher than in previous years, showing that the epidemic is not controlled.^1^ The World Health Organization (WHO) has identified high TB burden countries (HBC)^2^ and divided them into three categories: 22 HBCs, 41 HBCs that combine TB and HIV and 27 HBCs that also have high multidrug resistance. South Africa (SA) is one of the 14 countries that are listed in all three categories. Among the HBCs, SA has one of the highest prevalences of TB.^1^

The National TB Programme (NTP), since its inception in 1994, has shaped the South African response, with the three pillars of the programme being case finding, treatment and prevention.^3^ The introduction of Xpert^®^MTB/RIF to replace sputum smear microscopy in 2011 was a milestone for TB case finding.^4^ Improving TB treatment outcomes remains the biggest challenge. Treatment success rates among re-treatment cases are still poor, with high losses to follow-up of smear positive patients before initiating TB treatment, and high mortality rates even after successful treatment because of high TB–HIV co-infection rates.^5^

It is said that, ‘prevention of TB has been a neglected aspect of TB control’.^3^ The strategies in the NTP that impact on prevention include early diagnosis, early initiation of treatment and treatment of latent infection in high-risk groups. In 2006, SA adopted the WHO’s initiative of the ‘three I’s’ to reduce the burden of TB: isoniazid preventive therapy, intensified TB case finding and TB infection control (TBIC).^3^ The importance of infection control at health facility level was highlighted in SA after the description of the extreme drug-resistant (XDR) TB epidemic in 2006.^6,7^ The outbreak was characterised by a short incubation period between infection and disease, rapid deterioration and death within a few weeks.^6,7^ During this outbreak, about 90% of patients died within 4 months of the onset of symptoms.^6,7^ It was a wake-up call for SA and the rest of the world that nosocomial transmission of TB has deleterious consequences. In 2007, the NTP introduced TBIC as a specific programme, based on international recommendations (this document is still in draft form to the best of the authors’ knowledge).^8^

To understand the significance of potential nosocomial transmission of TB, a review of post-mortem diagnoses among hospitalised patients is needed. A post-mortem study from Zambia found that 62% of all deaths were because of undiagnosed TB.^9^ Another post-mortem study from SA showed 69% of early deaths on antiretrovirals (ARVs) were associated with undiagnosed TB.^10^ A study at Tygerberg Hospital concluded that 31 patients generated 345 inpatient infectious days with undiagnosed multidrug-resistant (MDR) TB during which other patients or staff could have been potentially infected.^11^ The concern around nosocomial TB transmission is not limited to other patients with compromised immunity, but is also a concern for health workers and other ancillary staff. Multiple studies from different countries show health care workers have a much higher risk of contracting TB than the general population.^12^

The need for a robust TBIC plan and practice at every health facility in SA is clearly justified. The NTP’s TBIC programme draft guideline has stipulated three main areas of infection control at health facilities: work practice and administrative control, environmental control focusing on the use of natural ventilation, and personal protection measures for health workers and other ancillary staff.^8^ Nine years after the introduction of the programme it is still unclear how much it has been implemented. Most of the audits of TBIC show limited implementation of the guideline.^13,14,15^

### Aim

The aim of this study was to identify the gaps and address the challenges in TBIC in the Mossel Bay sub-district in the Western Cape. The objectives were to assess the current quality of TBIC, plan and implement changes to improve the quality of TBIC, assess if the changes are associated with a measurable improvement in the quality of TBIC and develop local TBIC standard operating procedures.

### Setting

Mossel Bay sub-district is the second most populous area of the Garden Route District in the Western Cape. The sub-district has one district hospital, three primary health care (PHC) clinics, one community day care centre, six satellite clinics and four mobile clinics. Mossel Bay Hospital is a 90-bed district hospital which is divided into three general wards (male, female and paediatrics) and a maternity ward. Alma Clinic is located in the most populous area of the sub-district, with a rapid increase in formal and informal settlements. This PHC facility is particularly crowded and has a higher workload compared to other clinics in the sub-district. Alma Clinic is one of the three TB hotspot clinics outside of the Cape Town metropolitan area identified by the burden of disease project.^16^

## Methods

### Study design

**FIGURE 1:**
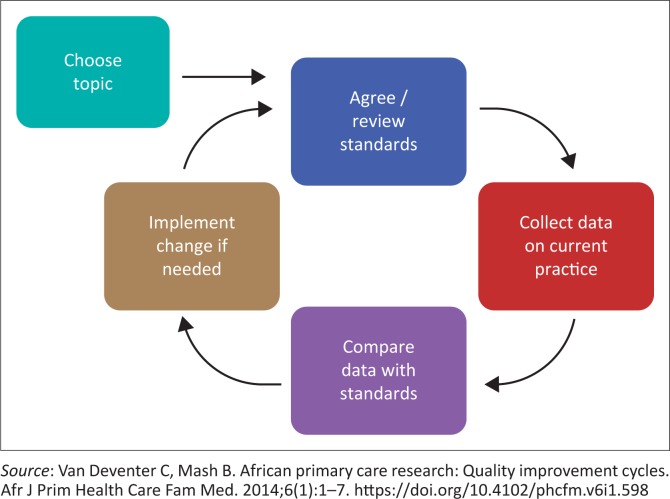
Steps of a quality improvement cycle.

This was a quality improvement study, composed of cycles and a series of steps (see Figure 1).^17^ The follow-up assessment was planned and performed 6 months after the baseline assessment.

### Selecting a team

The existing occupational health and infection control committee teams of the Mossel Bay Hospital and Alma Clinic were used as the quality improvement team for the study.

### Agreement on standards of tuberculosis infection control services

Both teams agreed with the 17 TBIC standards, as stipulated by the national TBIC draft guideline, to be the minimum criteria expected from the facilities as part of TBIC services.^8^

### Collect and analyse data on current practice and compare data with standards

#### Study population and sample size

The three main process criteria (criteria 4, 7 and 8 in Table 1) were evaluated using the before and after surveys of the proportion of patients visiting health facilities who were screened for TB and the mean time intervals between presenting with TB symptoms and sputum collection, as well as presenting with TB symptoms and initiation of therapy.

**TABLE 1:** National tuberculosis programme tuberculosis infection control target standards.

Variable		Means of verification†
**Workplace and administrative control**		
1	There is a written facility-specific infection control plan (that includes TBIC)	Document review
2	There is a designated person (and committee in larger facilities) responsible for implementing TBIC practices in the facility	Confirmation from facility manager
3	All clinical staff have received training or refresher training within the past 1 year on TBIC	Training records and IC nurse
4	All patients entering the facility are screened with a questionnaire. Those with a cough are given guidance on cough etiquette, separated from other patients and fast-tracked through all waiting areas, including consultation, investigations and drug collection	File review
5	Coughing patients are supplied with face mask and tissues	Observation in waiting areas
6	There is a tracking mechanism (e.g. register) and person responsible for monitoring turn-around time from TB screening to diagnosis and from TB diagnosis to treatment initiation	Review of the sputum register
7	The median time between screening positive for TB symptoms and actual diagnosis is no more than 1 day	File review
8	The median time between actual diagnosis and treatment initiation is no more than 1 day	File review
9	HIV testing is offered to all patients with presumptive TB and evaluation for time to start antiretroviral therapy (ART) is carried out if found HIV-positive	File review
**Environmental control**		
10	Waiting area is well ventilated (i.e. windows and doors open)	Site visit
11	Patients are not crowded in hallways or waiting areas	Site visit
12	Sputum samples are collected in a well ventilated, clearly designated area away from others, preferably outdoors	Presence of sputum collection area and observation
13	Diagnosed and suspected hospitalised TB patients are isolated or grouped according to sputum status in rooms with adequate natural ventilation	Site visit
**Staff protection**		
14	A package of HIV and HIV-associated TB prevention and care is available for facility staff on site including (1) confidential HIV testing and post-exposure prophylaxis for all staff, and (2) ART and isoniazid preventive therapy (IPT) for HIV+ staff	Occupational health nurse interview
15	Respirators are readily available for and being used by staff, particularly for high-risk aerosol-generating procedures and for providing care to patients with diagnosed or suspected infectious rifampicin-sensitive TB, MDR-TB and XDR-TB	Occupational health nurse interview
16	Staff have been trained in the proper fit and use of respirators	Occupational health nurse interview
17	TB symptoms occurring among staff are immediately investigated and, if TB is diagnosed, is treated, registered and reported in the confidential occupational health records or in the TB register	Occupational health nurse interview

TB, tuberculosis; TBIC, TB infection control; IC, infection control.

† , WHO checklist for periodic evaluation of TBIC.^18^

**Proportion of patients screened for tuberculosis (criterion 4):** This sample size was calculated using a *z*-test. To ensure a power of 80% to show a significant difference between two independent proportions (*p* < 0.05), a sample size of 152 patient records before and after the intervention was needed. Appreciating an attrition rate of 10%, a sample size of 165 patient records would be systematically reviewed before and after the intervention.

**The time intervals between patients presenting with TB symptoms and diagnosed with GeneXPert^R^ positive TB, and between positive sputum diagnosis and initiation of TB treatment (criteria 7 and 8):** The Wilcoxon–Mann–Whitney test was used to calculate this sample size. A sample size of 104 consecutive patient records before and after the intervention would give a power of 80% to show a statistically significant difference (*p* < 0.05), if the effect size = 40% (the researcher estimated a reduction in time interval from 5 to 2 days). Appreciating an attrition rate of 10%, a sample size of 110 patient records would be systematically reviewed before and after the intervention.

#### Data collection tools and process

A checklist of the standards of TBIC activities was completed after interviewing the occupational health and infection control persons of the two facilities at baseline and post-intervention. In addition, a record review was conducted for the three process criteria. The checklist was a modification of the WHO checklist for periodic evaluation of TBIC.^18^

**Proportion of patients screened for tuberculosis (criterion 4):** All patient records seen for 24 h of the day’s assessment were kept aside by the clerks and reviewed the same day or the day after the researcher completed an audit tool for each patient record reviewed. Data on demography, site where the patient was seen, category of the nursing personnel, working time, symptomatic TB screening and actions were collected.

**The time intervals between patients presenting with tuberculosis symptoms and diagnosed with GXP positive TB, and between positive sputum diagnosis and initiation of TB treatment (criteria 7 and 8):** The TB clinic nurses retrieved all the GXP positive TB patient records who were on treatment. The TB registers were used to identify the GXP positive patients. The duration used was dating back 6 months from the dates of the assessment. All consecutive patients’ records in that order were reviewed. The researcher completed an audit tool for each TB patient’s medical record reviewed. Data on demography, PHC clinic, time interval from presenting with TB symptom and sputum collection (interval 1); time interval from sputum collection to sputum result being available at the National Health Laboratory Services (NHLS) site (interval 2); and total time interval for presenting with TB symptoms to initiation of TB treatment in days (interval 3) were collected.

#### Data analysis

Quantitative data were captured in an MS Excel® spreadsheet, checked for omissions or errors and analysed in the Statistical Package for the Social Sciences (SPSS) software programme.^19^ The findings of the baseline assessment were summarised using simple descriptive statistics, and presented to the quality improvement cycle (QIC) team. Six months after implementing changes, the same data collection tools were used to assess any improvement in the quality of TBIC. Post-intervention data were added in the same data-capturing tool of the baseline. Pre- and post-intervention comparison was done for TB screening and time interval with inferential statistics using the chi-square test and Mann–Whitney *U* test to detect significant differences (*p* < 0.05).

### Feedback, planning and implementation of change

The findings of the baseline assessment for each site were presented to the respective teams. A reflection on the shortcomings of TBIC was facilitated via root cause analysis by asking consecutive ‘why?’ questions (e.g. a series of five consecutive ‘why?’ questions were initiated with the first question, ‘Why are windows not open in the wards over weekends and at night as per open window policy?’).^17^ As an outcome of this meeting, a set of action plans to improve the quality of TBIC were agreed upon (see Table 2). These action plans represent the intervention between the baseline and subsequent assessment. Members of the QIC team were delegated with specific tasks. The next date for reassessment was decided to be after 6 months.

**TABLE 2:** Feedback, planning and implementation of change for each site.

Site	Problems identified at baseline	Action plans agreed on (intervention)
Mossel Bay Hospital	Screening rate for TB was only 40% at OPD and EC. Poor compliance to the open window and door policy in the colder months and at night, otherwise very good adherence. Despite having open outside spaces for sputum collection for hospitalised patients, night staff collected sputum at bedside of patients. Sputum from symptomatic patients coming to EC was not always collected because staff believed sputum should be collected at local clinics, not at the hospital. Isolation rooms’ rules were not always respected by the staff. Isolation room was not available at EC, which was a challenge as some sick patients needed to stay in open EC setting for hours before isolation room bed was made available for them. Posters and signs were not adequately displayed around the hospital on cough hygiene, open window policy, and warning visitors that they were entering a high TB area with risk of acquiring infection.	Increased staff training on TBIC was conducted by the researcher, which included the nursing and supporting staff for each ward, as well as after-hour sessions for the night staff. The infection control person agreed to do daily rounds in every department to check for open window and door adherence and maintain an open window and door register. The infection control nurse met with the maintenance people to fix structural problems. The sub-district health office was approached to provide education materials (including posters on cough hygiene). Sputum specimen from ambulatory symptomatic patients was to be collected at EC any time of the day. Patients should be referred to the local clinic the next working day to access result and start treatment if tested positive. The medical manager of the sub-district would explore a plan to identify a temporary structure for isolating coughing sick patients waiting for admission from the EC.
Alma Clinic	The infection control and occupational safety committee was not active. The baseline assessment identified a good rate of TB screening (60%), but the quality of screening was questionable. Some of the waiting areas were extremely crowded and impossible to assure natural ventilation because of absence of windows and doors in these waiting areas. Some maintenance issues were found, including broken facemask dispensers, windows of consultation rooms unable to be opened, and the whirlybirds for roof ventilation. The median time to start TB treatment after presenting with TB symptoms for those who tested GXP positive was 4 days (96 h). The WHO recommendation is 48 h.	The facility’s operational manager committed to establish an active occupational safety and infection control committee. Continue to improve systematic TB screening services at all service points. This should be done as part of the initial observation for all patients seen at the facility. The staff will regularly survey the waiting crowd to identify coughing patients and fast-track them out of the clinic. One of the committee members agreed to be responsible for the open window and door register during a daily round. Another member would investigate and find out about the maintenance of the whirlybirds roof ventilation. Training was held for each members of the committee on the basics of TBIC by the researcher, with the plan that each member would train everyone else in their team. As natural ventilation was not possible in some of the waiting areas, the triage nurses will stagger the number of patients waiting in the poorly ventilated waiting areas by allowing only maximum four patients to sit and wait at those areas at a time. The aim was to reduce the time interval from presenting with TB symptoms to initiating therapy from 4 to 2 days, and to improve the working relationship with the CHWs and their coordinators.

TB, tuberculosis; OPD, outpatient department; EC, emergency centre; TBIC, TB infection control; CHWs, community health workers.

### Repeat analysis after intervention period and reflection on re-analysis

An assessment using the same checklist and the audit tool as the baseline was repeated 6 months after the intervention period, to document whether there was any improvement in TBIC practices. Medical records (a different set than the records used during the baseline audit) were audited to evaluate criteria 4, 7 and 8. Data were entered and analysed as in the baseline assessment described above.

### Ethical considerations

Informed voluntary consent was obtained from health workers who participated in the study. A waiver of informed consent was approved for data extracted from medical records and registers. Data were anonymised before analysis and reporting to preserve the confidentiality and privacy of individuals. Approval of the study was obtained from Higher Research Ethics Committee (reference number: S16/07/134) as well as permission from the Department of Health and local district managers (Provincial Health Research Committee reference number: WC_2016RP34_431).

## Results

The baseline and post-intervention samples of the data set used for the assessment of TB screening rate did not differ significantly with regard to some baseline characteristics like gender, age, race, triage category and time of the day the patients were seen.

### Workplace and administrative control

At baseline, many measures were already in place in both facilities to prevent nosocomial TB transmission, particularly administrative control. However, they were not optimally implemented. Mossel Bay Hospital had a specific TBIC policy. Alma Clinic also had a policy on infection control in general and a specific TBIC policy was compiled as part of the interventions suggested by the infection control committee during this intervention. At the hospital, there was a dedicated person for infection control and occupational safety, and an existing committee which met monthly (this was observed both at baseline and throughout the Quality Improvement cycle). However, at Alma Clinic the occupational safety and infection control committee existed only on paper at baseline as most of the members did not feel empower to own the role. During the implementation phase, meetings with facility and sub-district management lead to a revival of this clinic’s committee. The other measures in administrative control, like cough hygiene education to symptomatic patients, cough officers, fast tracing, making outdoors waiting area available for fast-tracked patients, providing masks to cover cough, were not measured quantitatively but were noted to be implemented inconsistently during the post-intervention assessment.

The quantitative assessment included the level of screening (criterion 4) at Mossel Bay Hospital and Alma Clinic. Mossel Bay Hospital OPD and EC had overall screening rate of 40% and Alma Clinic scored 61% at baseline. Post-intervention the screening rate dropped for Alma Clinic to 37% and at the Mossel Bay Hospital to 34.8% (see Table 3). The difference between the pre- and post-interventions was not statistically significant for Mossel Bay Hospital, but it was significant for Alma Clinic.

**TABLE 3:** Tuberculosis screening findings from baseline and post-intervention measurements.

Variable	Option	Baseline		Post-intervention		*p*
		*n*	%	*n*	%	
TB screening at Mossel Bay Hospital	Yes	34	40.0	40	34.8	0.271
	No	51	60.0	75	65.2	
TB screening at Alma Clinic	Yes	88	59.5	29	37.2	0.001*
	No	60	40.5	49	62.8	
TB screening combined for both sites	Yes	122	52.4	69	35.8	< 0.0001*
	No	111	47.6	124	64.2	
Result of screening	Positive	0	0.0	2	2.9	-
	Negative	121	100.0	67	97.1	

TB, tuberculosis.

* , Significant at *p* < 0.05

For the quantitative assessment of time intervals between patients presenting with TB symptoms and diagnosed as Expert^®^MTB/Rif positive, and between positive sputum diagnosis and initiation of TB treatment, 101 TB patients’ records were reviewed from Alma Clinic before and after the intervention. The 101 patients represent the total population of sputum Expert^®^MTB/Rif positive patients who were receiving care at Alma Clinic. The baseline and post-intervention samples of the data set used for the assessment of time intervals between printing with TB symptoms and initiation of treatment did not differ significantly with regard to some baseline characteristics like gender, age, race, and presence of co-morbidities. No statistically significant differences in intervals were found between baseline and post-intervention assessments (see Table 4). The median time for a patient presenting with TB symptoms until initiation of TB treatment once sputum was Expert^®^MTB/Rif positive was 4 days at baseline. This delay did not show improvement post-intervention and remained the same. The time interval between presenting with TB symptoms and sputum collection, and the time from sputum collection to when sputum results were available on the NHLS system, was almost always 1 day. Interval 3 (the time interval between when sputum result was available in the NHLS system until the initiation of TB treatment) was where most of the delay occurred, contributing to the overall 4-day delay from presenting with TB symptoms until the starting of TB treatment (see Table 4).

**TABLE 4:** Time interval between presenting with tuberculosis symptoms and initiating tuberculosis therapy (*N* = 101 for pre- and post-intervention, Alma Clinic).

Variable	Baseline (days)	Post-intervention (days)	*p*
Median	4	4	0.959 (retain the null hypothesis)
Range	58	30	-

### Environmental control

Environmental control is limited to respecting the open window and door policy and having a functioning whirlybirds roof ventilation. During the baseline and follow-up assessment, all consultation and ward rooms had open doors and windows. Mossel Bay Hospital has open door and window register completed by the Occupational Health and Safety (OHS) nurse on her daily round. However, this did not happen consistently in cold weather and at night. At Alma Clinic the team could not locate the open door and window register during the intervention period. The register was introduced at the time of the baseline assessment. There was no dedicated person completing the register despite assigning the task to one of the infection control committee members. Assessing the appropriate functioning of the roof ventilators was a challenge at the baseline as there were no technical persons in the team. Later, maintenance staff confirmed that none of the roof ventilators were in working condition because of lack of maintenance. Alma Clinic had some permanent infrastructure challenges at the time of the QI cycle. Some of the waiting areas and the emergency room were devoid of windows and doors opened into a corridor with no direct communication with the outside space.

### Staff protection

All measures to protect health care workers and staff were in place. The IC and OHS nurses at each site were open and available for any staff member who needed the service. It was up to the discretion of health care workers to access these services at the occupational health and safety clinic. N-95 masks were consistently available everywhere and their appropriate utilisation was the responsibility of every health worker. Fits tests were conducted regularly.

## Discussion

This QIC aimed to evaluate and improve the TBIC, against a national draft policy document within a high TB burden, rural sub-district of the Western Cape Province. The QI cycle involved two facility types within the sub-district, the district hospital and one of its main PHC clinics. Each facility had an existing Infection and Prevention Control and OHS team, which were used as the audit teams. A planned set of interventions following the baseline assessment did not show significant improvement in the implementation of the national TBIC policy document components (workplace and administrative control, environmental control and staff protection).

Looking at the process indicators, TB screening and the time delay before initiation of TB treatment, there was no objective improvement comparing before and after intervention at both facilities. This was complicated by the fact that the baseline assessment at Alma Clinic was not valid as it documented an exceptionally high TB screening rate which was biased because of the knowledge of the nurses about the reason for the assessment on the day of the baseline data collection (the so-called Hawthorne effect).^20^ This deterioration in practice was verified by the proportion of Xpert^®^MTB/RIF patients identified with the TB screening tool, which dropped from 26% at the baseline to 1% post-intervention.

It was hoped that the first cycle of the TBIC QIC would result in developing robust TBIC standard operating procedures for Mossel Bay sub-district, in line with the national policy document. On the one hand, this cycle managed to clarify the questions to be asked and which interventions to prioritise. For example, there was a need to focus on administrative control at both facilities as this was controlled by health workers and could be improved without additional resources. On the other hand, this first cycle unmasked several potential reasons for the delay between diagnosis and start of treatment which required further unpacking. A similar delay of 4 days was documented by Schmidt et al. who compared the difference before and after Xpert^®^MTB/RIF rollout on parameters related to TB diagnosis.^21^ After the introduction of Xpert^®^MTB/RIF, the median time from presenting with TB symptoms to initiation of TB treatment was reduced form 5 days to 4 days.^21^ This begs the question of how realistic the WHO target of 48 h is for SA. The extreme outliers on the baseline assessment at Alma Clinic were discussed with the TB clinic nurse to explore the reason for the long delay before initiation of treatment. There was good documentation of the efforts made by the TB nurse and community health worker (CHW) in trying to contact the patients, but the biggest challenges were incorrect or absent addresses and incorrect telephone numbers.

Most of the anticipated improvements following the implementation of interventions were dependent on the health workers’ adherence to the locally agreed TBIC policies. While exploring factors affecting health workers’ compliance with policies was not one of the study objectives, it emerged as a significant factor. For example, systematic TB screening is not a complicated task and the tool required was available at the desk of each health worker. Each health worker was trained in screening, but it was difficult to explain why it was not done. The importance of buy-in and capacitating health workers to engage with the QI process was supported by Shah et al., who described the ways in which health workers appraise their own behaviour.^22^ These authors reported that health workers experienced an ‘ambiguity about responsibility of certain infection control practices’, a ‘divergence in values attached to some infection control practices by prioritising other activities’ and a ‘hierarchy of influence’, which prevented health workers from fully engaging on these matters as they found it difficult when they perceived that their superiors were not really respecting and supporting the policies practically.^22^ This phenomenon on hierarchy of influence was observed in the QI cycle during the TBIC training of hospital-based nurses as they were asking if doctors also knew the TBIC guidelines, as many of the activities required the participation of doctors. It was also noted at Alma Clinic that the IC and OHS team did not feel empower to be able to do what they were assigned to do because of conflicting policy decisions from their seniors. The best baseline finding for Alma Clinic was the open waiting area of the TB clinic and that the TB clinic was physically separated from the rest of the clinic structure, which made it easier to implement TBIC measures. This also enabled coughing patients to wait in an open waiting area at the TB clinic while waiting to provide sputum specimens. This separation is supported by the TBIC standard as described on the draft national guideline on TBIC.^8^ However, during the intervention period a decision was made by management to move the TB clinic into the building where the rest of chronic illness and acute care clinics were run. This brought in patients with various types of TB, including MDR-TB, to wait in the same waiting area as the acute and chronic care patients. This situation arose from implementing two conflicting policy guidelines for clinics, namely, the ‘National Core Standards’,^23^ which is in favour of separation of patients with TB, and the ‘Ideal clinic’,^24^ which states that patients with TB are part of chronic illness and should be integrated with the rest of the chronic illness clinic. This apparent ambiguity in policy directives was echoed by Zinatsa et al. in their recent qualitative study, ‘Voices from the frontline: barriers and strategies to improve tuberculosis infection control in primary health care facilities in South Africa’.^25^ Clarity is required on how to ensure TBIC when integrating chronic care services at PHC facility level. What put health care workers at high-risk of nosocomial infection was not the lack of staff protection measures but the poor implementation of administrative and environmental control measures.

### Limitations

The baseline assessment of TB screening at Alma Clinic was prejudiced because of the knowledge of nurses as to the purpose of the baseline assessment. This was noticed in retrospect and during the intervention period as routine TB screening was not as enthusiastic as that of the baseline. This made us question the validity of the baseline screening assessment at Alma Clinic as a potential source of bias was introduced at the time of data collection and only retrospectively noted. Another limitation was the over ambitiousness of the project which aimed at targeting all 17 standards. The success of the project depended on consistent implementation of multiple activities, and it was observed over time that the teams lost focus. The lead researcher, as family medicine registrar, felt challenged by the realities of the context which influenced her ability to play a consistent role as facilitator of the quality improvement initiative, especially at the PHC facility level. The aspired roles of the doctor in the PHC facility have been agreed upon recently by a set of national stakeholders, which aimed to challenge the current reality in which primary care doctors have a perceived limited influence beyond merely performing a clinical outreach function.^26^ This reality of having to ‘push the numbers’^27^ (service delivery priority with little or no role in strengthening the service) may have impacted on the registrar’s availability to help drive the TBIC QIC on an ongoing basis. Lastly, the lack of representation of maintenance and infrastructure personnel in the clinic team limited implementation as challenges of maintenance and structure were constant themes in every meeting of the IC and OHS team.

### Recommendations

The study showed that one QIC is not adequate to resolve implementation challenges and the need to make ongoing QICs an integral part of facilities’ work, with periodic evaluation. Next steps should include an ongoing QIC, which focuses on specific areas of TBIC. There is an urgency to focus on administrative control, specifically TB screening, as this is the first line of defence against nosocomial TB transmission. Having knowledge about what needs to be done for TBIC does not necessarily translate into consistent behavioural change (the so-called ‘know-do gap’). Further research in the local context should focus on creating a better understanding of the factors which enable implementation of the policy.

## Conclusion

The aim of this study was to assess and improve the quality of TBIC in the Mossel Bay sub-district. The study found good managerial commitment reflected by the presence of various policies, guidelines, specific personnel and committees to deal with infection control in general. The project has created awareness about TBIC among the staff. The project also pointed out the complexity of health workers’ behaviour towards adhering to policies.

## References

[1] World Health Organization Global tuberculosis control: WHO report 2018 [homepage on the Internet]. Geneva: World Health Organization; 2018 [cited 2019 Jan 11]. Available from: https://www.who.int/tb/publications/global_report/en/.

[2] Technical Advisory Group for TB (STAG-TB) Use of high burden country lists for TB by WHO in the post-2015 era discussion paper [homepage on the Internet]. World Health Organization; 2015 [cited 2016 Feb 27]. Available from: http://www.who.int/tb/publications/global_report/high_tb_burdencountrylists2016-2020.pdf.

[3] Churchyard GJ, Mametja LD, Mvusi L, et al Tuberculosis control in South Africa: Successes, challenges and recommendations. S Afr Med J. 2014;104(3):234–248. 10.7196/SAMJ.7689 24893501

[4] National Department of Health National strategic plan on HIV, STIs and TB: 2012–2016 [homepage on the Internet]. [cited 2019 Jan 15]. Available from: http://www.doh.gov.za/docs/stratdocs/2012/NSPfull.pdf.

[5] Claassens MM, du Toit E, Dunbar R, et al Tuberculosis patients in primary care do not start treatment: What role do health system delays play? Int J Tuberc Lung Dis. 2013;17(5):603–607. 10.5588/ijtld.12.0505 23575324

[6] Gandhi NR, Weissman D, Moodley P, et al Nosocomial transmission of extensively drug-resistant tuberculosis in a rural hospital in South Africa. J Infect Dis. 2013;207(1):9–17. 10.1093/infdis/jis631 23166374 PMC3523793

[7] Bateman C Tugela Ferry’s extensively drug-resistant tuberculosis-10 years on. S Afr Med J. 2015;105(7):517–520. 10.7196/SAMJnew.7838 26447248

[8] Department of Health of South Africa The draft national infection prevention and control policy for TB, MDRTB and XDRTB [homepage on the Internet]. Pretoria: Department of Health of South Africa; 2007 [cited 2019 Jan 15]. Available at: http://www.who.int/hiv/pub/guidelines/south_africa.pdf.

[9] Bates M, Mudenda V, Shibemba A, et al Tuberculosis at post-mortem in inpatient adults at a tertiary referral centre in sub-Saharan Africa – A prospective descriptive autopsy study. Int J Mycobacteriol. 2015;4:75–76. 10.1016/j.ijmyco.2014.10.054 25765217

[10] Wong EB, Omar T, Setlhako GJ, et al Causes of death on antiretroviral therapy: A post-mortem study from South Africa. PLoS One. 2012 Oct 16;7(10):e47542 10.1371/journal.pone.0047542 23094059 PMC3472995

[11] Bamford CM, Taljaard JJ Potential for nosocomial transmission of multidrug-resistant (MDR) tuberculosis in a South African tertiary hospital. S Afr Med J. 2010 Jul;100(7):438–441. 10.7196/SAMJ.3501 20822590

[12] McCarthy KM, Scott LE, Gous N, et al High incidence of latent tuberculous infection among South African health workers: An urgent call for action. Int J Tuberc Lung Dis. 2015;19(6):647–653. 10.5588/ijtld.14.0759 25946353

[13] Engelbrecht MC, Van Rensburg AJ Tuberculosis infection control practices in primary healthcare facilities in three districts of South Africa. S Afr J Epidemiol Infect. 2013;28(4):221–226. 10.1080/10158782.2013.11441554

[14] Malangu N, Mngomezulu M Evaluation of tuberculosis infection control measures implemented at primary health care facilities in Kwazulu-Natal province of South Africa. BMC Infect Dis. 2015;15(1):1 10.1186/s12879-015-0773-7 25887523 PMC4369348

[15] Mphahlele MT, Tudor C, van der Walt M, Farley J An infection control audit in 10 primary health-care facilities in the Western Cape Province of South Africa. Int J Infect Cont. 2012;8(3):1–5.

[16] Draper B, Pienaar D, Parker W, Rehle T Recommendations for policy in the Western Cape Province for the prevention of major infectious diseases, including HIV/AIDS and tuberculosis [homepage on the Internet]. Cape Town; 2007 [cited 2018 June 14]. Available from: https://vula.uct.ac.za/access/content/group/91e9e9d8-39b6-4654-00ae-f4d74cba085f/CD%20Volume%203%20%20Major%20Infectious%20Diseases%20HIV%20T.doc.

[17] Van Deventer C, Mash B African primary care research: Quality improvement cycles. Afr J Prim Health Care Fam Med. 2014;6(1):1–7. 10.4102/phcfm.v6i1.598 PMC4502901 26245438

[18] World Health Organization Checklist for periodic evaluation of TB infection control in health-care facilities [homepage on the Internet]. Geneva: World Health Organization; 2015 [cited 2015 Mar 17]. Available from: http://www.who.int/tb/areas-of-work/preventive-care/checklist_for_periodic_evaluation_of_tb_infection_control_in_health_facilities.pdf.

[19] IBM Corp Released 2017. IBM SPSS Statistics for Windows, Version 25.0. Armonk: IBM Corp; 2017.

[20] McCambridge J, Witton J, Elbourne DR Systematic review of the Hawthorne effect: New concepts are needed to study research participation effects. J Clin Epidemiol. 2014;67(3):267–277. 10.1016/j.jclinepi.2013.08.015 24275499 PMC3969247

[21] Schmidt BM, Geldenhuys H, Tameris M, et al Impact of Xpert MTB/RIF rollout on management of tuberculosis in a South African community. S Afr Med J. 2017 Dec;107(12):1078–1081. 10.7196/SAMJ.2017.v107i12.12502 29262960

[22] Shah N, Castro-Sánchez E, Charani E, et al Towards changing healthcare workers’ behaviour: A qualitative study exploring non-compliance through appraisals of infection prevention and control practices. J Hosp Infect. 2015;90:126–134. 10.1016/j.jhin.2015.01.023 25820128

[23] National Department of Health ‘Towards quality care for patients’: National core standards for health establishments in South Africa. Pretoria, South Africa: National Department of Health; 2011.

[24] Steinhobel R, Massyn N, Peer N The Ideal Clinic Programme 2015/16. Durban: Health Systems Trust; 2015.

[25] Zinatsa F, Engelbrecht M, van Rensburg AJ, Kigozi G Voices from the frontline: Barriers and strategies to improve tuberculosis infection control in primary health care facilities in South Africa. BMC Health Serv Res. 2018;18(1):269 10.1186/s12913-018-3083-0 29636041 PMC5894140

[26] Mash R, Malan Z, Von Pressentin K, Blitz J Strengthening primary health care through primary care doctors: The design of a new national Postgraduate Diploma in Family Medicine. S Afr Fam Pract. 2015;58(1):32–36.

[27] Moosa S, Gibbs A A focus group study on primary health care in Johannesburg Health District: ‘We are just pushing numbers’. S Afr Fam Pract. 2014;56(2):147–152. 10.1080/20786204.2014.10855353

